# *Lycium Barbarum* (Wolfberry) Reduces Secondary Degeneration and Oxidative Stress, and Inhibits JNK Pathway in Retina after Partial Optic Nerve Transection

**DOI:** 10.1371/journal.pone.0068881

**Published:** 2013-07-19

**Authors:** Hongying Li, Yuxiang Liang, Kin Chiu, Qiuju Yuan, Bin Lin, Raymond Chuen-Chung Chang, Kwok-Fai So

**Affiliations:** 1 Department of Anatomy and the State Key Laboratory of Brain and Cognitive Science, Li Ka Shing Faculty of Medicine, The University of Hong Kong, Hong Kong, China; 2 Research Centre of Heart, Brain, Hormone and Healthy Aging, Li Ka Shing Faculty of Medicine, The University of Hong Kong, Hong Kong, China; 3 GMH Institute of Central Nervous System Regeneration, Jinan University, Guangzhou, China; Schepens Eye Research Institute, Harvard Medical School, United States of America

## Abstract

Our group has shown that the polysaccharides extracted from *Lycium barbarum* (LBP) are neuroprotective for retinal ganglion cells (RGCs) in different animal models. Protecting RGCs from secondary degeneration is a promising direction for therapy in glaucoma management. The complete optic nerve transection (CONT) model can be used to study primary degeneration of RGCs, while the partial optic nerve transection (PONT) model can be used to study secondary degeneration of RGCs because primary degeneration of RGCs and secondary degeneration can be separated in location in the same retina in this model; in other situations, these types of degeneration can be difficult to distinguish. In order to examine which kind of degeneration LBP could delay, both CONT and PONT models were used in this study. Rats were fed with LBP or vehicle daily from 7 days before surgery until sacrifice at different time-points and the surviving numbers of RGCs were evaluated. The expression of several proteins related to inflammation, oxidative stress, and the c-jun N-terminal kinase (JNK) pathways were detected with Western-blot analysis. LBP did not delay primary degeneration of RGCs after either CONT or PONT, but it did delay secondary degeneration of RGCs after PONT. We found that LBP appeared to exert these protective effects by inhibiting oxidative stress and the JNK/c-jun pathway and by transiently increasing production of insulin-like growth factor-1 (IGF-1). This study suggests that LBP can delay secondary degeneration of RGCs and this effect may be linked to inhibition of oxidative stress and the JNK/c-jun pathway in the retina.

## Introduction

Glaucoma has been considered to be a neurodegenerative disease characterized by optic nerve (ON) atrophy and irreversible loss of retinal ganglion cells (RGCs) [1]. The loss of RGC bodies may be primary (caused by direct damage to axons or cell bodies, such as crush or transection of axons) or secondary (caused by the toxious effectors released from the neighboring dying cells because of primary damage or a cell death signal from the deafferented target) [2]–[5]. The delay of secondary degeneration of RGCs in glaucoma is believed to provide a promising avenue for treatment.

Several animal models have been used in the study of glaucoma, including complete optic nerve transection (CONT), acute and chronic ocular hypertension models and the ON crush model. However, it is difficult to distinguish primary degeneration from secondary degeneration in these commonly used models because each involves insult to all RGCs [3]. For example, in the CONT model, all the axons of RGCs are cut and therefore all RGCs will die from primary degeneration. However, in the partial optic nerve transection (PONT) model, which was established about ten years ago, only axons in the dorsal part of ON are transected. The degeneration of the cell bodies of RGCs whose axons are transected during surgery is primary and the degeneration of the cell bodies of RGCs whose axons are intact during surgery is secondary. According to the literature, primary degeneration mainly happened in superior retinas and secondary in inferior retinas, they could be separated in location. [2]. Oxidative stress has been thought to be involved in secondary degeneration after PONT, even though stringent measures are taken to ensure adequate retinal circulation [6]–[8]. Inflammation has also been shown to be involved in secondary degeneration after brain trauma and spinal cord injury. However, its involvement in secondary degeneration of RGCs after PONT has not been studied.

*Lycium barbarum* has been used as an “upper class herb” for hundreds of years in the Oriental world. It was used for the treatment of the diseases related to vision, the “kidney” and the “liver” [9]. We have shown that the polysaccharides extracted from *Lycium barbarum* (LBP) reduce the death of cultured cortical neurons challenged by beta-amyloid, glutamate and Homocysteine [10]–[13]. LBP also delay the degeneration of RGCs in a rat chronic ocular hypertension model [14] and a mouse acute ocular hypertension model [15] and reduce neuronal damage in a mouse transient middle cerebral artery occlusion model [16]. However, it is difficult to know whether LBP delayed primary or secondary degeneration in these models, and the mechanism or mechanisms underlying the neuroprotective effects of LBP for neuronal tissues remained unclear.

The aims of this experiment were to confirm whether ON section caused retinal oxidative stress, to investigate the presence of retinal inflammation after ON section and to determine which kind of degeneration LBP could delay and which mechanism(s) might be involved in any neuroprotective effects of LBP, and we were largely successful in these aims.

## Materials and Methods

### Ethics Statement

The use of animals followed the requirements of the Cap. 340 Animals (Control of Experiments) Ordinance and Regulations in Hong Kong. All the experimental and animal handling procedures were approved by the Faculty Committee on the Use of Live Animals in Teaching and Research in The University of Hong Kong (CULATR #1850-09 and #1996-09).

### Animals and Procedure

**Figure 1:**
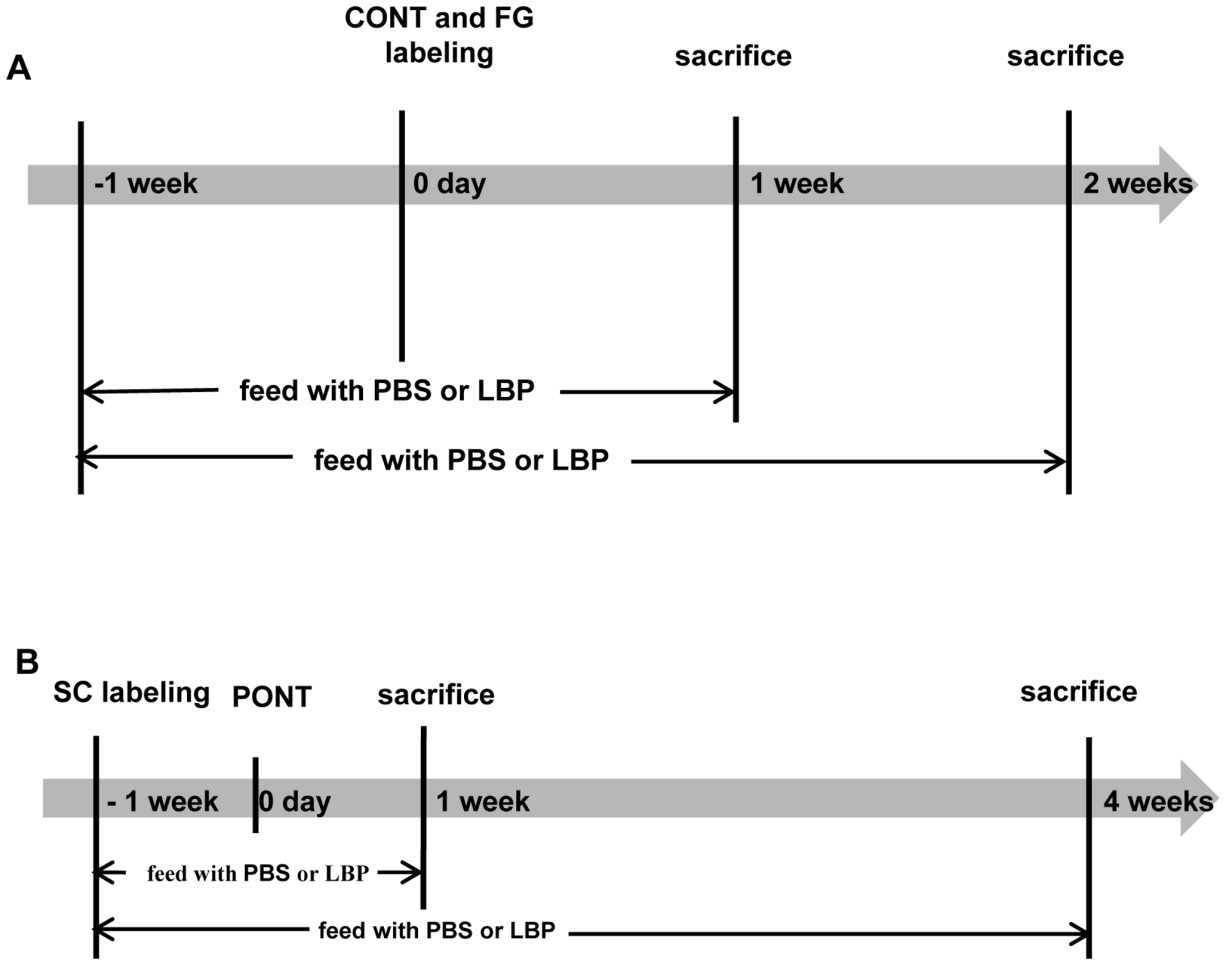
Schematic diagrams showing the procedures for the estimation of RGC survival. Rats were fed with PBS or LBP 1 week before CONT or PONT until sacrifice. (A) In CONT experiments, CONT was performed and then a piece of gelatin soaked with FG was placed close to the ON stump to label RGCs on day 0. Rats were sacrificed 1 week or 2 weeks after surgery. (B) In PONT experiments, SC labeling was performed 1 week before PONT and rats were sacrificed 1 week or 4 weeks after surgery.

Adult female Sprague Dawley rats (10–12 weeks of age weighing 250–280 g) were used in this study. The rats were housed in a temperature-controlled room subjected to a 12-hour light/12-hour dark cycle and supplied with food and water *ad libitum*. The preparation of LBP was as previously described [8]. The final powder was stored in a dry-box and freshly dissolved in phosphate - buffered saline (PBS; 0.01 M; pH 7.4) before use. The treatment (LBP or PBS) began 1 week before surgery (CONT or PONT) until sacrifice at the scheduled time-points (see Fig. 1). The treatment was achieved with a feeding needle by gavage once daily.

To investigate if the degeneration speeds were similar between superior and inferior retinas after CONT, the rats without treatment with PBS or LBP were sacrificed either 1 week or 2 weeks after CONT (n = 5 at either time-point). To evaluate the effects of LBP on the survival of RGCs after ON injury, the procedure was as described in Fig. 1. There were 4 to 16 animals in each group: CONT: n = 10, 8, 16 and 12 in PBS, 0.1 mg/kg LBP, 1 mg/kg LBP and 10 mg/kg LBP groups sacrificed 1 week after CONT. n = 7 and 6 in PBS and 1 mg/kg LBP groups sacrificed 2 weeks after CONT. PONT: n = 7 and 4 in PBS and LBP groups sacrificed 1 week after PONT. n = 9 and 10 in PBS and LBP groups sacrificed 4 weeks after PONT.

Retrograde labelling of RGCs was achieved using Fluoro-Gold (FG) from the stump of the ON after CONT [17] or from superior colliculi (SC) 1 week before PONT [18]. Seven rats without treatment or ON injury were sacrificed 7 days after SC labeling as controls for both CONT and PONT experiments. Eight animals were used for 1, 1′-dioctadecyl-3, 3, 3′, 3′-tetramethylindocarbocyanine perchlorate (DiI) tracing *in vivo*. The death of cells in ganglion cell layer (GCL) was studied using terminal deoxynucleotidyl transferase-mediated dUTP-biotin nick end labeling assay (TUNEL assay) and protein expression was examined with Western-blot analysis at 12 hours, 1 day, 4 days and 1 week after PONT; there was no drug treatment (n = 3 to 5 animals in each group).

Protein expression after LBP or PBS treatment was also studied with Western-blot analysis both 1 day and 1 week after PONT (n = 3 to 5 animals in each group). The rats for RGC counting (both after FG and DiI labeling) and Western-blot analysis were sacrificed using inhalation of CO_2_. For TUNEL assay, the rats were sacrificed by injecting overdoses of Phenobarbital followed by perfusion with 0.9% NaCl and 4% paraformaldehyde (PFA).

### Surgical Procedure

Anesthesia and the CONT procedure were conducted as previously described [17]. The PONT surgery was similar to that described by Fitzgerald et al [8]. The partial incision in the ON was made 1.0 mm from the optic disc and was achieved using a pair of Spring Vannas scissors (15000-08, F.S.T., Heidelberg, Germany) marked 200 µm from the tips of both blades, or using a diamond knife (G-31480, Geuder AG, Hertzstrasse, Heldelberg, Germany) with the blade fixed to a length of 200 µm.

### Retrograde DiI Tracing *in vivo* after PONT

**Figure 2:**
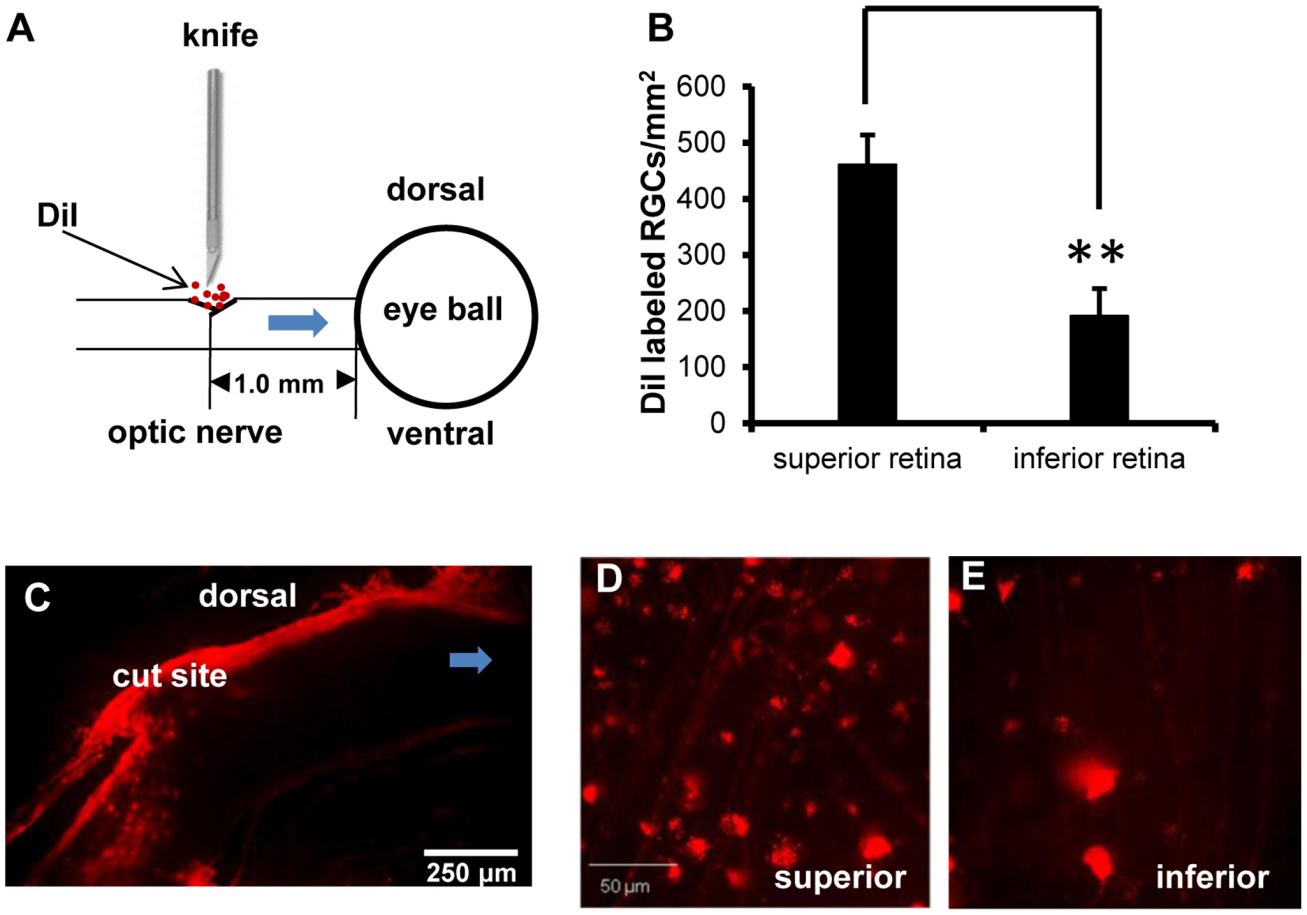
RGCs and ON labeled with DiI *in vivo*. (A) Schematic diagram of DiI labeling: after partial incision in the dorsal ON 1.0 mm from the optic disc, several crystals of DiI were precisely placed into the incision. The DiI were transported to the retinas via the ends of the axons attached to the eyeball. (B) More RGCs in the superior retinas were labeled with DiI than in the inferior retinas (Student *t*-test, **P = 0.001). (C) DiI labeled axons were limited to the dorsal part of the ON. (D) Photographs about 1 mm from the optic disc in both the superior and inferior retinas showed the different densities of DiI labeled RGCs. (n = 8).

The method published by Fitzgerald et al. was adopted [8]. Briefly, the ON was partially cut and several crystals of DiI (Molecular Probes, Eugene, OR) were placed precisely into the cut sites to label the RGCs whose axons were transected (Fig. 2A). The rats were sacrificed 4 days after DiI labeling. The retinas were processed for RGC counting as below.

Optic nerves were collected, post-fixed in 4% PFA for 60 minutes and then placed into 30% sucrose in 0.1 M phosphate buffer solution overnight until they sank. They were then embedded into optimal cutting temperature embedding compound and sectioned longitudinally. The sections were mounted on slides, rinsed 3 times with 0.01 M PBS and observed using fluorescence microscopy.

### Quantification of RGCs

**Figure 3:**
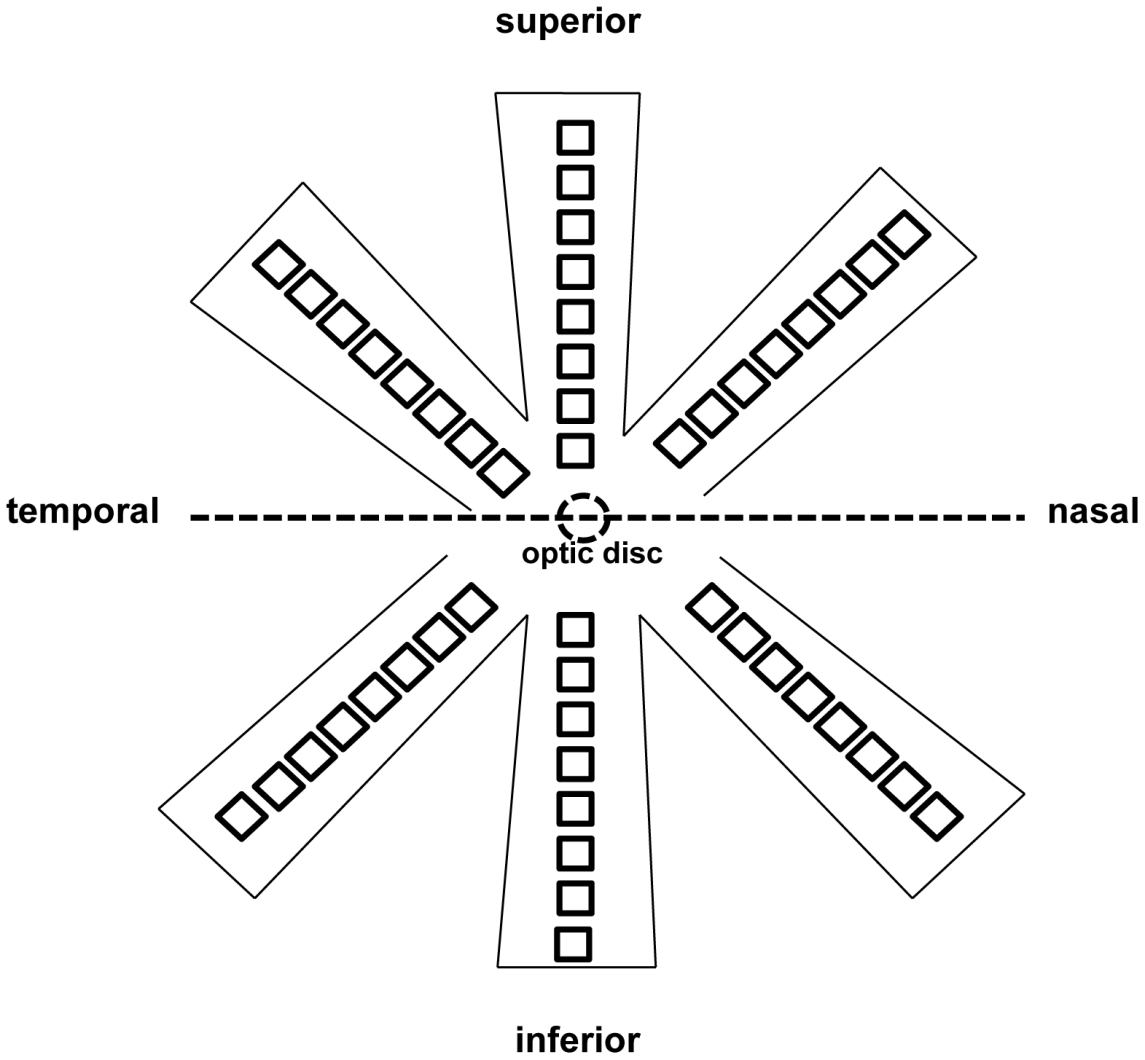
Schematic diagram showing the method for taking photographs for RGC counting. Retinas were divided into the superior and inferior halves and each half was separated into three sectors (temporal, middle, nasal) before being flat-mounted. Eight photographs each 200×200 µm^2^ were taken along the median line of each sector, starting from the optic disc to the border at 500 µm intervals. A total of 48 photographs per retina were taken.

After sacrifice, retinas were collected and post-fixed in 4% PFA for 60 minutes. Retinas were divided into the superior and inferior halves and each half was separated into three roughly equal sectors before being flat-mounted as the temporal, middle and nasal sectors (Fig. 3). Eight photographs (200×200 µm^2^) in each sector were captured along the median line, starting from the optic disc to the edges at 500-µm intervals under a fluorescence microscope at 400×magnification [14], [19]. The limitation of using photographs for cell counting rather than focusing through whole-mounted retinas is that under-estimation may occur. However the counting method is unlikely to alter the results of this experiment and this method has the merit that the photographs can be kept longer than sections and be recounted. Using rats without treatment with PBS or LBP, we showed similar RGC surviving densities between superior and inferior retinas either 1 week or 2 weeks after surgery (see Results). Therefore, for rats treated with PBS or LBP, only inferior retinas were used after CONT. After PONT, surviving RGCs were counted separately in superior and inferior retinas because the degeneration speeds were different in superior and inferior retinas after PONT [2], and grouped together for the whole retinas. The counting was conducted by a double-blind method by two persons and the data were averaged (mean ± SEM, numbers per mm^2^).

### TUNEL Assay

TUNEL staining was previously believed to detect apoptosis only, but more recently it has been shown to detect necrosis and other types of cell death as well [20]. To determine when cell death begins in the GCL, TUNEL staining was used to examine retinas at different time-points after PONT. After sacrifice, the eyeballs were post-fixed in 4% PFA overnight at 4°C, dehydrated with a graded series of ethanol and xylene, and then embedded in paraffin. Cross-sections (4 µm) were cut using a microtome (Micro HM 315R, Heidelberg, Germany). Manufacturer’s instructions for the TUNEL assay were followed (Roche Diagnostics GmbH, Mannheim, Germany). Sections were counterstained with 4′, 6-diamidino-2-phenylindole (DAPI) after the TUNEL reaction to confirm that the TUNEL staining was located in the nuclei. For consistency of analysis, only sections with ON head were selected for observation. Three sections were selected from each animal. The positive-staining cells in the GCL in the inferior retinas were counted under a microscope using a 400×magnification. The data were expressed as mean ± SEM, numbers per inferior retina.

### Western-blot Analysis

After sacrifice, the inferior retinas were collected in PBS on ice. The procedure including the use of lysis buffer, secondary antibody and the developing reagents were as previously described [17], [19]. After transfer onto polyvinylidene difluoride membrane, the membranes were blocked with 5% non-fat dry milk or 3% bovine serum albumin in Tris-buffered saline with 0.05% Tween 20 for 1 hour. Rabbit polyclonal antibody tumor necrosis factor alpha (TNF-α, 1∶500), mouse monoclonal antibody manganese superoxide dismutase (MnSOD or SOD2, 1∶4000) and rabbit polyclonal antibody brain-derived neurotrophic factor (BDNF, 1∶100) were supplied by Abcam (Cambridge, MA, USA). Goat anti-mouse polyclonal antibody insulin-like growth factor 1 (IGF-1, 1∶500) was purchased from R&D System (Minneapolis, USA). Rabbit polyclonal antibodies phospho-c-jun N-terminal kinases (p-JNKs, 1∶500) and phospho-c-jun (p-c-jun, 1∶500) were purchased from Cell Signaling Technology (Beverly, MA, USA). The tissue was incubated in Tris-buffered saline with 0.05% Tween 20 overnight at 4°C. The incubation of secondary antibody (dilution 1∶10000 for MnSOD and 1∶2000 for others) was conducted for 1 hour at room temperature. Protein loading was controlled using the monoclonal mouse antibody against α-tubulin (1∶20000, Sigma-Aldrich, St. Louis, MO, USA). Densitometric analysis was performed using Image J software (National Institutes of Health, USA) with the scanned autoradiographic films.

### Statistical Analysis

Student’s *t-*test was used for comparisons of two groups. For more than two groups, one-way ANOVA was used for multiple comparisons followed by Dunn’s or Student-Newman-Keuls method as *post hoc* tests. Data were analyzed statistically with the Sigmastat software (Sigmastat 3.5; Systat Software Inc., Chicago, IL, USA). The P = 0.05 level was considered to be statistically significant.

## Results

### RGCs Degenerated Significantly after CONT and PONT

**Figure 4:**
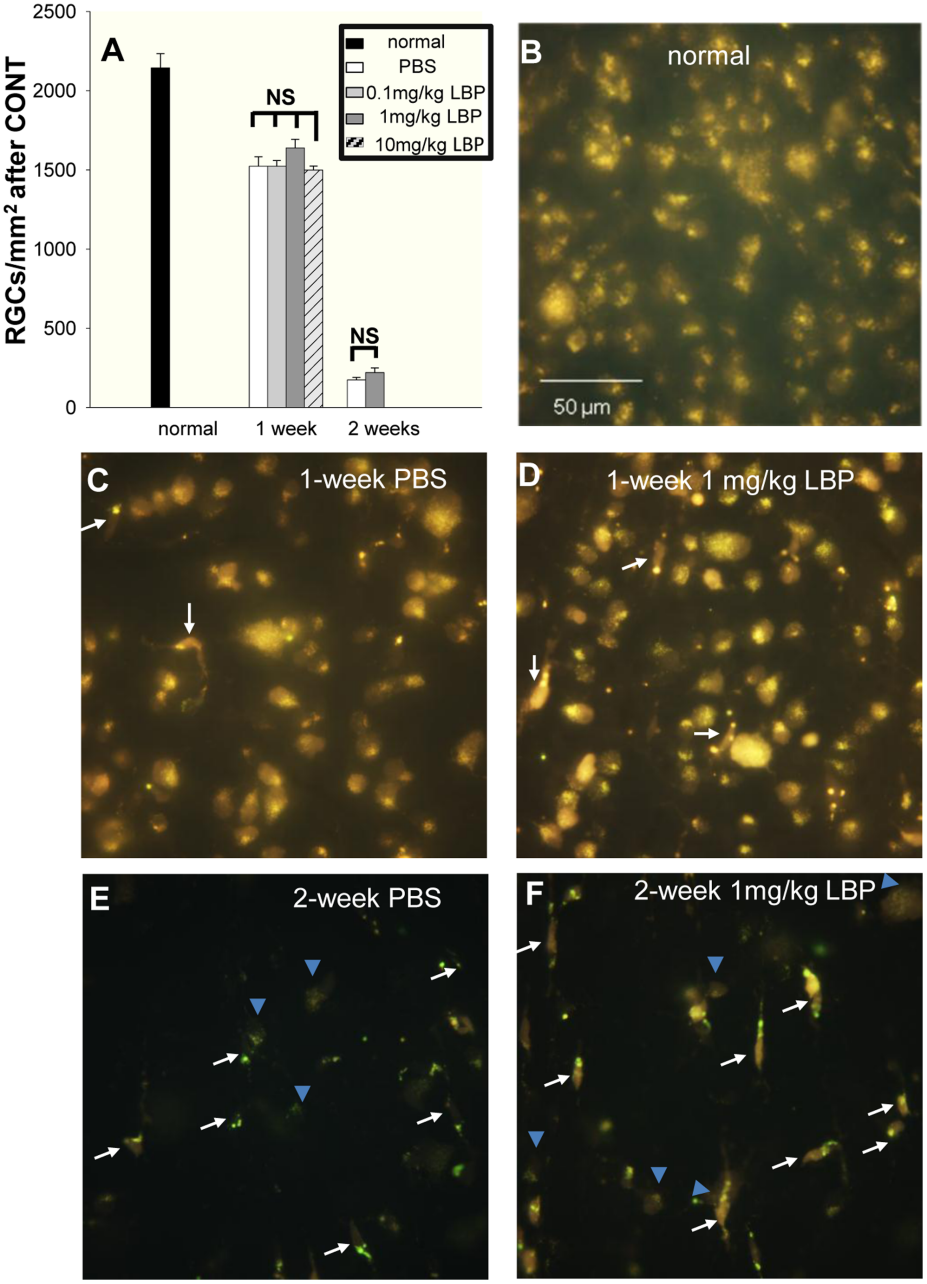
Effects of LBP on survival of RGCs 1 week and 2 weeks after CONT. RGCs were labeled by FG. The arrows indicate microglia which were easily distinguished from RGCs and not counted. The blue arrowheads indicate RGCs. (A, C, D) Orally feeding of 0.1 mg/kg, 1 mg/kg and 10 mg/kg LBP showed no significant effects on the survival of RGCs 1 week after CONT (compared with PBS group) and no significant difference among the three different dosages of LBP groups was detected. (A, E, F) 1 mg/kg LBP showed no significant effects on the survival of RGCs 2 weeks after CONT (compared with PBS group). (n = 10, 8, 16, 12 in PBS, 0.1 mg/kg LBP, 1 mg/kg LBP, 10 mg/kg LBP groups sacrificed 1 week after CONT and n = 7 and 6 in PBS and 1 mg/kg LBP groups sacrificed 2 weeks after CONT.).

**Figure 5:**
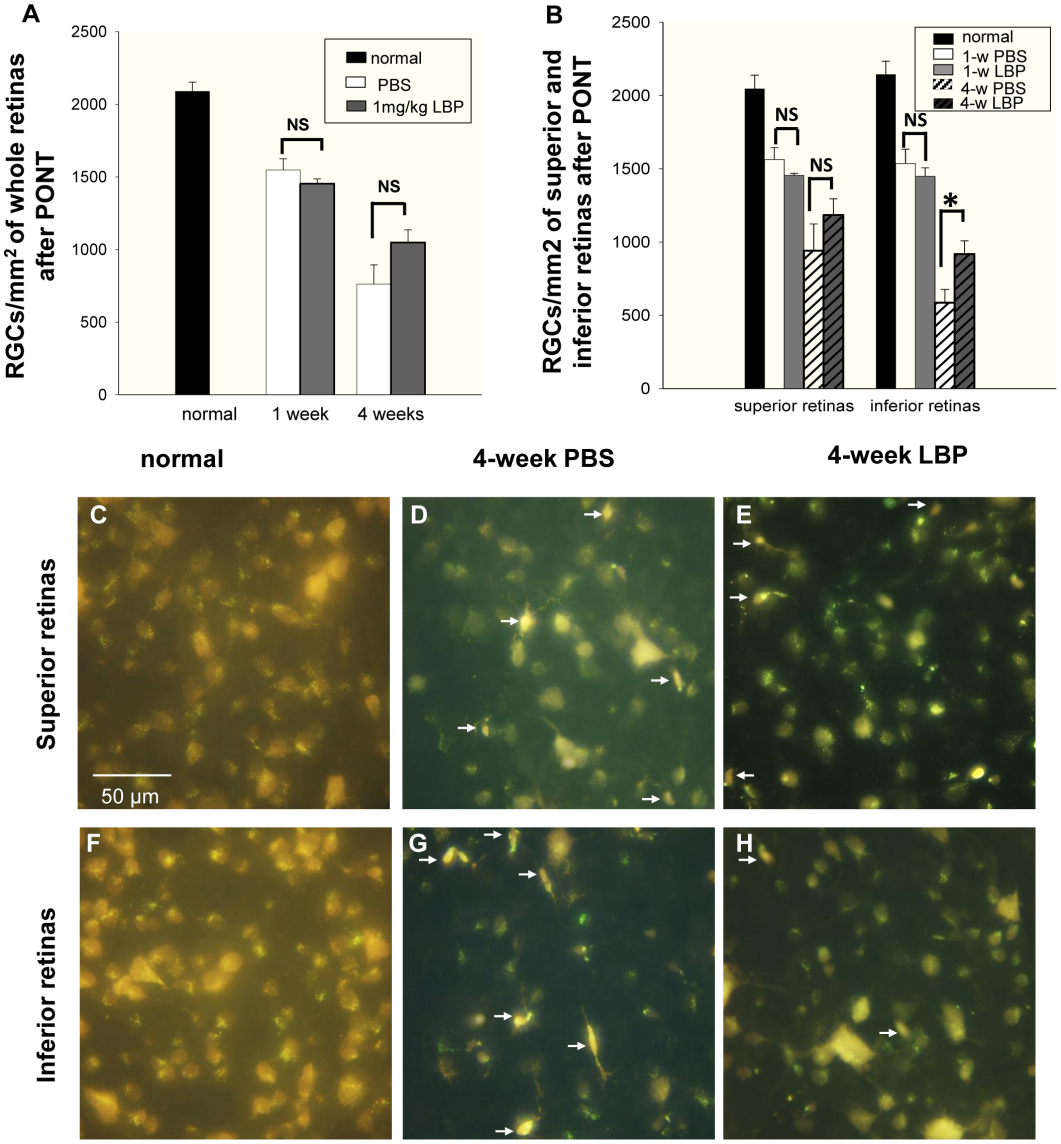
Effects of LBP on RGC survival 1 week and 4 weeks after PONT. The RGCs were labeled with FG. (A) LBP did not increase the survival of RGCs either 1 week or 4 weeks after the PONT when the densities of surviving RGCs were produced from the whole retinas (NS: not significant). (B) When the retinas were divided into the superior and inferior halves, LBP did not delay the degeneration of RGCs 1 week after PONT. However, it reduced the degeneration of RGCs in the inferior retina (*P = 0.027) but not in the superior retina 4 weeks after the PONT. (F – H) The photographs of RGCs labeled by FG in both the superior and inferior retinas are about 1.5 mm away from the optic disc. In the superior retinas, the densities of RGCs were similar between the PBS and LBP groups. In the inferior retinas, the density of RGCs in the LBP group was higher than that in the PBS group. Microglia (white arrows) were easily distinguished from RGCs and not counted. (n = 7 and 4 in PBS and LBP groups 1 week after PONT. n = 9 and 10 in PBS and LBP groups 4 weeks after PONT.).

The average densities of FG-labeled RGCs in the normal retinas were as follows: the whole retinas: 2088.1±64.4 RGCs/mm^2^; the normal superior retinas: 2046.5±92.4 RGCs/mm^2^; and the normal inferior retinas: 2144.4±89.8 RGCs/mm^2^. There was no difference between the superior and inferior retinas (Student’s *t*-test, P>0.05). The surviving RGC densities decreased significantly in the expected areas after both CONT and PONT in animals treated with PBS or LBP (Student’s *t*-test, P<0.001, Fig. 4 & Fig. 5). The surviving densities of RGCs after CONT from animals without treatment with PBS or LBP were as follows: 1510.7±65.6 in the superior retinas and 1402.6±74.7 in the inferior retinas 1 week after CONT; 234.2±19.8 in the superior retinas and 214.8±8.4 in the inferior retinas 2 weeks after CONT. There were no significant differences between the superior and inferior retinas at both time-points after CONT (Student’s *t*-test, P>0.05).

### LBP did not Prevent the Primary Degeneration of RGCs after CONT

One PBS group and three LBP groups with different dosages (0.1 mg/kg, 1 mg/kg and 10 mg/kg) were examined 1 week after CONT. No significant difference between the PBS group and any LBP group was detected; in addition, no significant difference among the three dosages of LBP was seen (one-way ANOVA for multiple comparisons and Dunn’s method as post hoc tests: Fig. 4A, 4C & 4D). We have previously found that 1 mg/kg LBP can significantly reduce the death of RGCs 2 weeks and 4 weeks after ocular hypertension produced by laser photocoagulation [14], and therefore 1 mg/kg LBP was adopted in the later experiments (CONT 2 weeks and PONT). Two weeks after CONT, no significant difference between PBS and LBP groups was detected (Student’s *t*-test, P>0.05, Fig. 4A, 4E & 4F).

### LBP Delayed Secondary Degeneration of RGCs in the Inferior Retina 4 Weeks after PONT

DiI labeled the cell bodies of RGCs whose axons were transected after PONT and which would be expected to die from primary degeneration. There were 460.9±52.8 RGCs/mm^2^ and 191.2±48.7 RGCs/mm^2^, labeled in the superior and inferior retinas respectively. The difference was significant (P = 0.001, Fig. 2B, 2D & 2E) and the ratio was about 2.4∶1 between superior and inferior retinas. These findings indicate that both superior and inferior retinas are vulnerable to primary and secondary degeneration after PONT. However, in the inferior retinas, significantly more RGCs would be affected by secondary injury since the inferior retina has significantly fewer RGCs with axons transected by PONT surgery.

LBP had no effect on the survival of RGCs in whole retinas either 1 week or 4 weeks after PONT; comparison of the PBS and LBP groups showed no difference between groups at either time-point (Fig. 5A). When dividing the retinas into superior and inferior halves, there was no difference in the superior retinas between PBS and LBP groups either 1 week or 4 weeks after PONT (Fig. 5B, 5D & 5E). LBP protected about 18% of RGCs in the inferior retinas 4 weeks after the PONT but not 1 week after PONT (one way ANOVA, p<0.05, Fig. 5B, 5G & 5H).

Combining the results from DiI labeling and the survival of RGCs, our data show that LBP appears to delay secondary degeneration of RGCs rather than to affect primary degeneration.

### DiI Labeled Axons Located in the Dorsal ON

The sections from the optic nerves with retrograde labeling of the RGCs by DiI showed that the travel path of DiI from the cut site to the retinas was limited to the dorsal part of the nerve (Fig. 2C).

### Oxidative Stress and JNK Pathway(s) Involved in Degeneration of RGCs in the Inferior Retina after PONT

**Figure 6:**
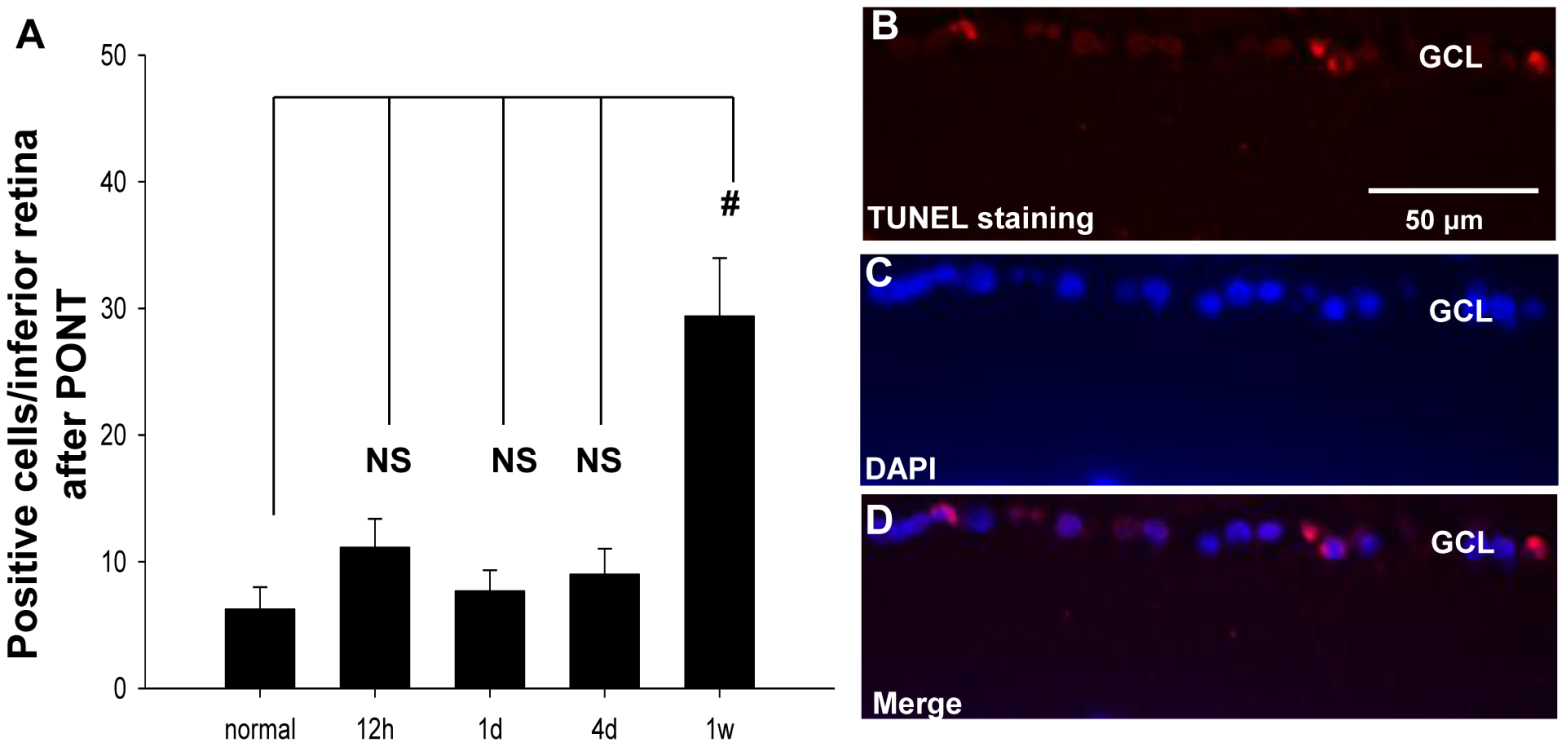
TUNEL staining in the inferior retinas after PONT. (A) The number of positive-staining cells increased significantly in the inferior retinas 1 week after PONT, compared with the normal retinas (P<0.01). There were no significant differences between normal and any other group. (B) The positive cells in the GCL of the inferior retina 1 week after PONT (red). The positive staining was located in the nuclei (C, D), which is shown with DAPI (blue). (n = 4, 3, 3, 5 and 4 in normal, 12 h, 1d, 4d and 1w groups respectively.).

In the inferior retinas, TUNEL staining showed that the number of positive-staining cells increased significantly 1 week after PONT (one way ANOVA, P<0.01). However, there were no changes at 12 hours, 1 day and 4 days. The positive staining was shown in the nuclei, which was confirmed by counter-staining with DAPI (Fig. 6).

**Figure 7:**
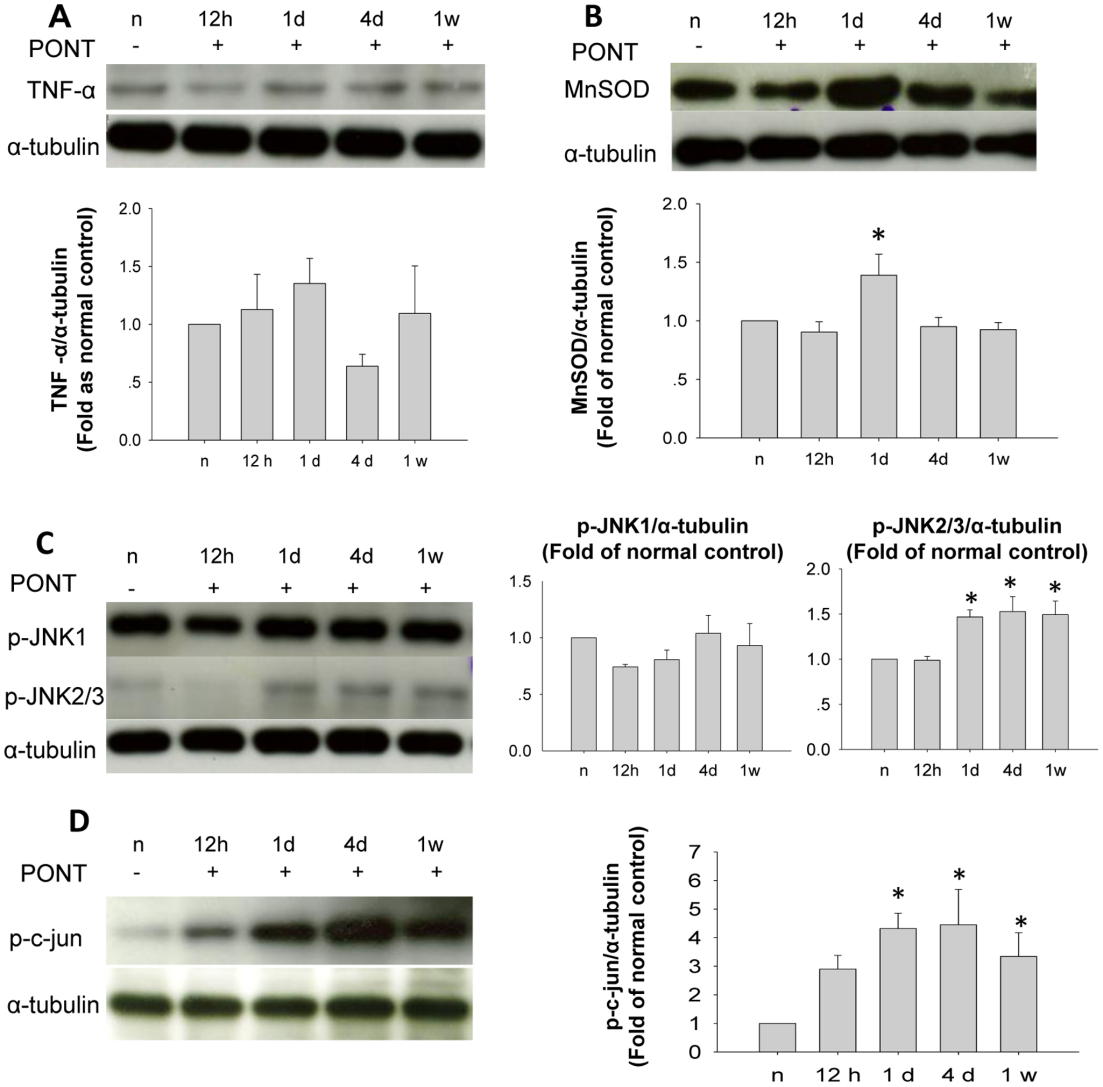
Western-blot analysis of the inferior retinas after PONT. Each band from one animal. (A) The expression levels of TNF-α in the inferior retinas did not change after PONT at different time-points (P>0.05). (B) MnSOD level increased significantly 1 day after PONT and had returned to normal 4 days after PONT (*P<0.05) (C) P-JNK1 level did not change after PONT (P>0.05). P-JNK2/3 levels increased significantly from 1 day until 1 week after PONT (*P<0.05). (D) The levels of p-c-jun increased from 1 day until 1 week after PONT (*P<0.05). (n = 3 in each group.).

The protein level of TNF-α did not increase after PONT in the inferior retinas (Fig. 7A). The expression of MnSOD increased significantly 1 day after PONT and returned to normal level 4 days after PONT (Fig. 7B). The p-JNK/p-c-jun pathway was also involved in the degeneration of RGCs in the inferior retinas. Although the expression of p-JNK1 did not change, the level of p-JNK2/3 increased 1 day after PONT and was maintained until 1 week (Fig. 7C). P-c-jun increased with the same tendency as p-JNK2/3 (Fig. 7D).

### LBP Inhibited Oxidative Stress and Activation of JNK Pathway as well as Transiently Increasing the Expression of IGF-1 in the Inferior Retina

**Figure 8:**
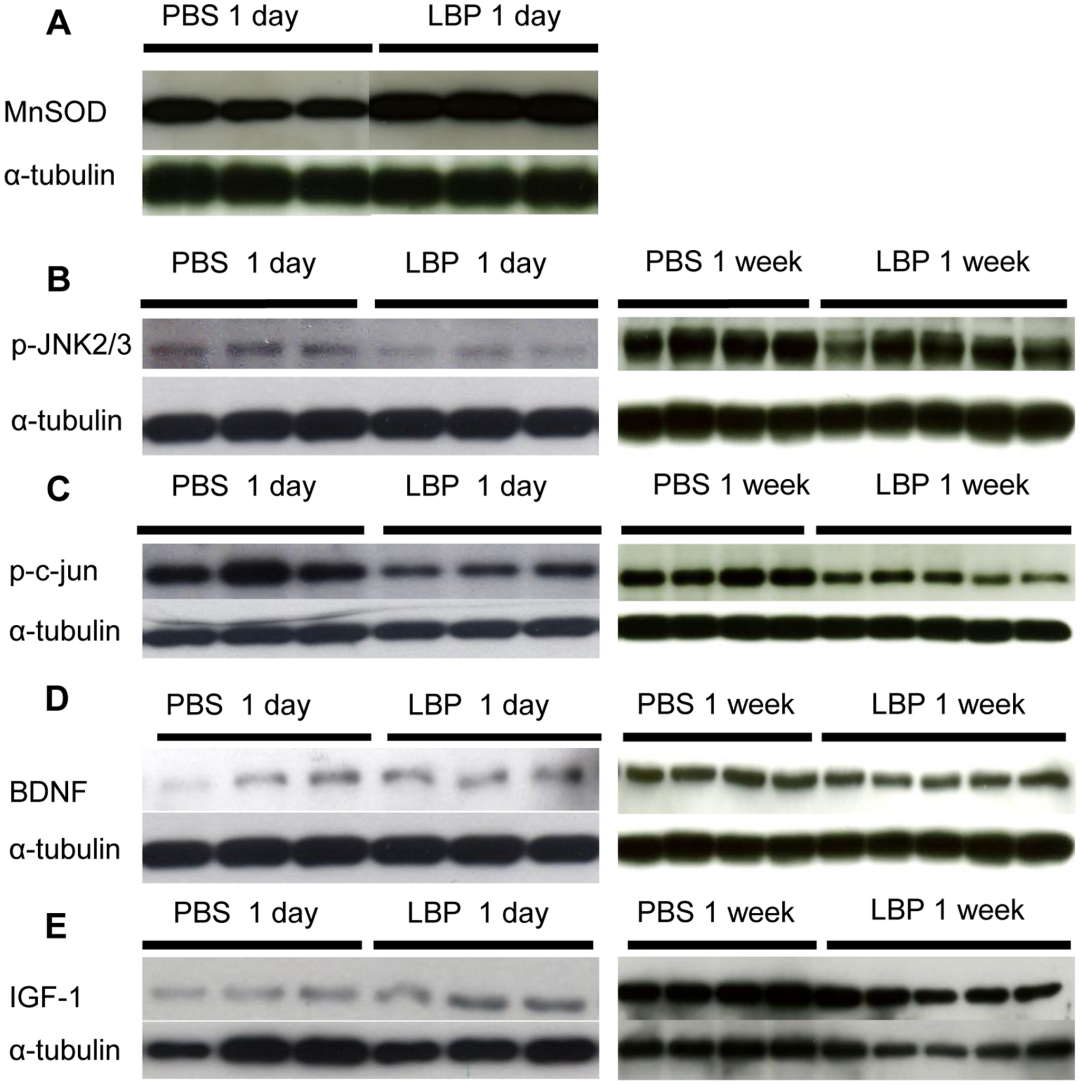
Western-blot analysis of the inferior retinas after treatment with PBS or LBP. Each band is from one animal. (A) The expression of MnSOD 1 day after PONT both in PBS and LBP groups. (B, C, D, E) The expressions of p-JNK2/3, p-c-jun, BDNF, IGF-1 both 1 day and 1 week after PONT both in PBS and LBP groups. (n = 3, 3, 5 and 4 in PBS 1 day, LBP 1 day, PBS 1 week and LBP 1 week groups respectively.).

**Figure 9:**
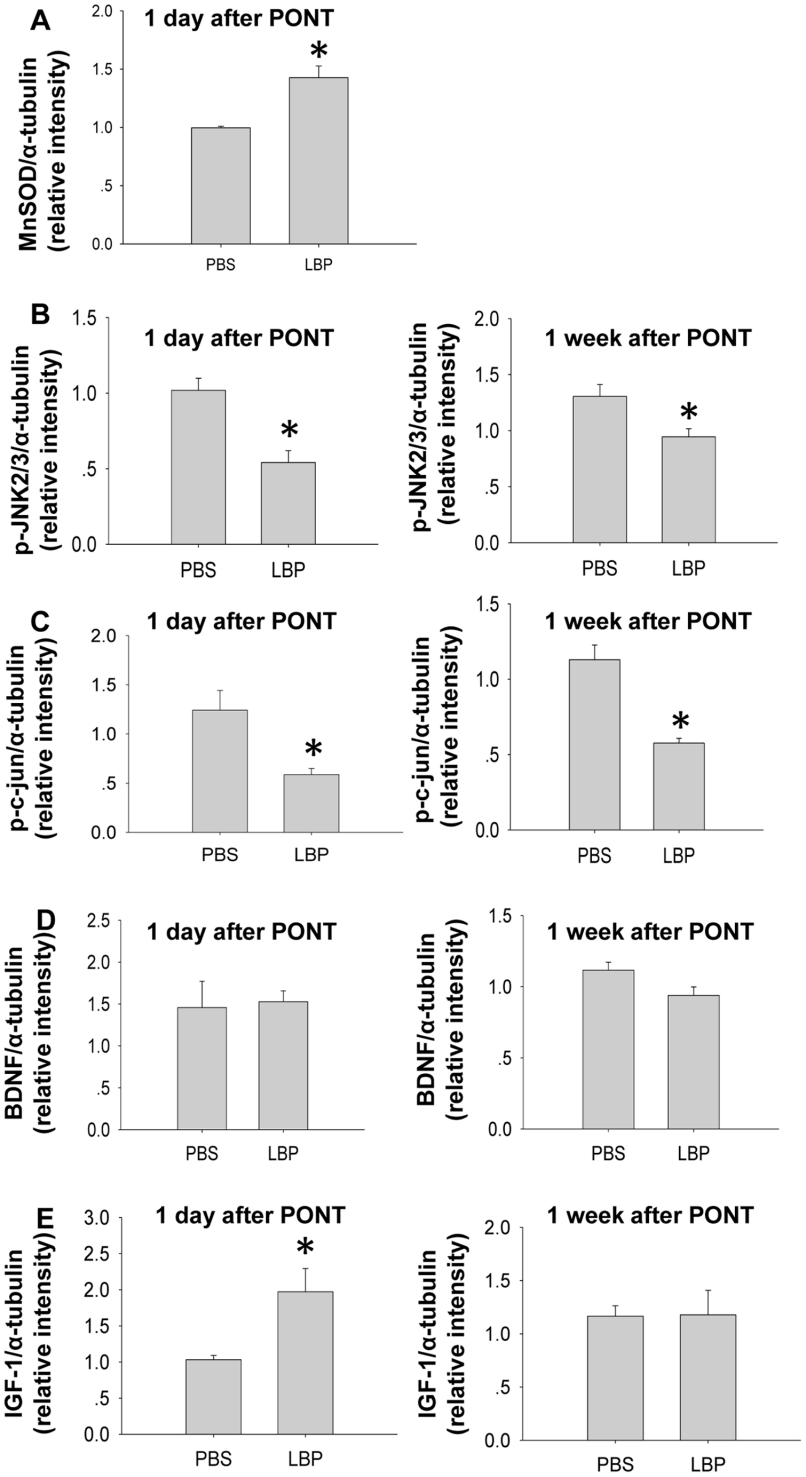
Western-blot analysis of the inferior retinas after treatment with PBS or LBP. (A) LBP increased the expression of MnSOD 1 day after PONT (*P<0.05). (B, C) LBP decreased the expression of p-JNK2/3 and p-c-jun (*P<0.05). (D) LBP did not change the expression of BDNF either 1 day or 1 week after PONT (P>0.05). (E) LBP increased the expression of IGF-1 1 day after PONT (*P<0.05), but did not change the expression of IGF-1 1 week after PONT (P>0.05). (n = 3, 3, 5 and 4 in PBS 1 day, LBP 1 day, PBS 1 week and LBP 1 week groups respectively.).

After LBP treatment, the expression of MnSOD increased significantly 1 day after PONT (Fig. 8A & Fig. 9A). On the other hand, LBP treatment significantly decreased the expression of p-JNK2/3 and p-c-jun both 1 day and 1 week after PONT (Fig. 8B, 8C & Fig. 9B, 9C). The effects of LBP on the expression BDNF and IGF-1 were as follows: after PONT, LBP did not change the expression of BDNF either 1 day or 1 week after PONT (Fig. 8D & Fig. 9D). However, LBP increased the expression of IGF-1 1 day after PONT, but the effect was not maintained at 1 week (Fig. 8E & Fig. 9E).

## Discussion

After CONT, most RGCs died rapidly from primary degeneration. After PONT, more RGCs die from secondary degeneration at a later time-window in addition to primary degeneration [2], [6]. Our results showed that LBP did not delay primary degeneration of RGCs after CONT. However, LBP did delay secondary degeneration of RGCs 4 weeks after PONT. Levkovitch-Verbin et al. showed that although the genetic profile was similar for primary and secondary degeneration of RGCs, minocycline was only effective for secondary degeneration, indicating a potential difference between the two types of degeneration [21]. Our result was consistent with this in that LBP only delayed secondary degeneration but not primary degeneration.

In the PONT model, the increasing expression of MnSOD or SOD2, which was demonstrated by immunohistochemistry (IHC), was used as an indicator of oxidative stress [7], [22], [23]. MnSOD is an anti-oxidant enzyme and can detoxify in cells and tissues by converting toxic superoxide into hydrogen peroxide and diatomic oxygen. Administration of adeno-associated virus containing the SOD2 gene into eyes significantly reduces oxidative stress and nitrative stress in a rat acute ocular hypertension model [24]. The protective effect of LBP for RGCs was related to the anti-oxidative mechanism [19]. In order to determine if the anti-oxidant ability of LBP for RGCs was related to MnSOD, we investigated the expression levels of MnSOD in the rats treated with LBP or vehicle, and our results confirmed the anti-oxidant effect of LBP in retinas after injury.

JNKs are the kinases involved in both apoptotic and non-apoptotic cell death [25], [26]. C-jun is a transcription factor activated by phosphorylation of JNKs and is involved in the transcription of various proteins, including some pro-apoptotic proteins [26]. Previous studies using the PONT model and IHC staining have shown that JNKs are activated at the primary injury sites and c-jun is activated both at the primary and the secondary injury sites in the retina [7], [27]. There are three isoforms of JNKs: JNK1, JNK2 and JNK3; IHC cannot differentiate among these isoforms. We used Western-blot analysis, to differentiate JNK1 from JNK2/3 according to the molecular weights. Our results confirmed the inhibition of the JNK/c-jun pathway by LBP; this effect has been shown previously using different models [28], [29]. However, this is the first time that these effects of LBP have been demonstrated in the retina. In addition, our results showed that p-JNK2/3 rather than p-JNK1 were activated in the inferior retina after PONT. A similar result has been shown in cultured RGC-5 cells: advanced glycation end products–albumin from bovine serum increased the production of p-JNK2/3, but not p-JNK1 *in vitro* [30].

BDNF belongs to the neurotrophin family and is expressed both in SC [31], [32] and retina [33]. The level of BDNF increases in retina following ON transection [34] and after periocular injection of *in situ* hydrogels containing Leu-Ile. This is an inducer for neurotrophic factors, which increase the expression of BDNF in the retina and promote RGC survival after ON injury [35]. IGF-1 is also a neurotrophic factor which is a key molecule determining the survival of RGCs during the early stage of ON injury [36]. However, the effects of LBP on the expression of BDNF and IGF-1 have not been previously studied. Our results show that LBP can produce a transient increase in the expression of IGF-1 in the inferior retina, but the source of this IGF-1 is not clear. Future study using IHC with this model may help to address this issue.

It is known that DiI could be transported by either active processes or by diffusion [6], [37]–[40]. In this experiment, DiI was used to label RGCs whose axons were transected after PONT. Although it has been reported that DiI can label cells in close proximity to labeled cells in fixed tissues [37], this phenomenon has not been reported *in vivo* [40]. Perhaps the time available for DiI labeling for fixed tissues was much longer than that *in vivo*; diffusion to neighboring tissue was obvious in fixed tissues but not for the tissues *in vivo*. Therefore, we did an *in vivo* study where diffusion of DiI was limited. Our results also showed that the axonal transport of DiI was limited to the dorsal region in the ON sections from the labeled animals and where diffusion was insignificant.

Our results confirmed the neuroprotective effects of LBP for RGCs and showed the possible mechanism. The future target of our study is to provide the basis for the use of LBP in clinical conditions. The electroretinogram is used widely by ophthalmologists and optometrists for the diagnosis of retinal diseases and can evaluate the retinal function by measuring the electrical responses of various cell types [41]–[45]. Therefore, we have also used the electroretinogram to evaluate the effect of LBP after PONT and this experiment is currently in process.
